# Development of Robust Cationic Light-Activated Thermosensitive Liposomes: Choosing the Right Lipids

**DOI:** 10.1021/acs.molpharmaceut.3c00602

**Published:** 2023-10-24

**Authors:** Puja Gangurde, Mohammad Mahmoudzadeh, Zahra Gounani, Artturi Koivuniemi, Patrick Laurén, Tatu Lajunen, Timo Laaksonen

**Affiliations:** † Drug Research Program, Division of Pharmaceutical Biosciences, Faculty of Pharmacy, University of Helsinki, Viikinkaari 5 E, FI-00790 Helsinki, Finland; ‡ School of Pharmacy, University of Eastern Finland, P.O. Box 1627, FI-70211 Kuopio, Finland; § Faculty of Engineering and Natural Sciences, Tampere University, FI-33101 Tampere, Finland

**Keywords:** cationic liposomes, light activation, thermosensitive, stimuli-responsive release, membrane fluidity, molecular dynamics simulations

## Abstract

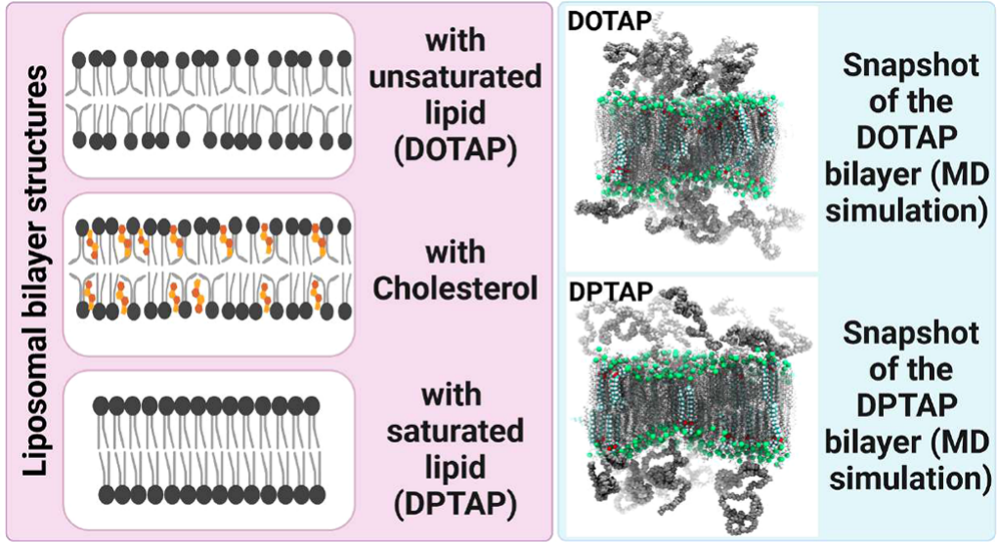

Extensive research has been conducted on cationic light-activated thermosensitive liposomes (CLTSLs) as a means for site-specific and controlled drug release; however, less attention has been given to the stability of these nanoparticles. Selecting the appropriate lipids is crucial for the development of a stable and responsive system. In this study, we investigated the impact of various lipids on the physical properties of cationic light-activated liposomes. Incorporating poly(ethylene glycol) PEG molecules resulted in uniform liposomes with low polydispersity index, while the addition of unsaturated lipid (DOTAP) resulted in extremely leaky liposomes, with almost 80% release in just 10 min of incubation at body temperature. Conversely, the inclusion of cholesterol in the formulation increased liposome stability too much and decreased their sensitivity to stimuli-responsive release, with only 14% release after 2 min of light exposure. To achieve stable and functional CLTSL, we substituted an equivalent amount of unsaturated lipid with a saturated lipid (DPTAP), resulting in stable liposomes at body temperature that were highly responsive to light, releasing 90% of their content in 10 s of light exposure. We also conducted two atomistic molecular dynamics simulations using lipid compositions with saturated and unsaturated lipids to investigate the effect of lipid composition on the dynamical properties of the liposomal lipid bilayer. Our findings suggest that the nature of lipids used to prepare liposomes significantly affects their properties, especially when the drug loading needs to be stable but triggered drug release properties are required at the same time. Selecting the appropriate lipids in the right amount is therefore essential for the preparation of liposomes with desirable properties.

## Introduction

Liposomes have demonstrated their effectiveness in delivering targeted anticancer drugs, leading to reduced toxicity in healthy tissues and improved therapeutic efficacy of encapsulated drugs.^1−3^ Liposomes can efficiently deliver chemotherapeutics to tumors due to their long circulation half-life and reduced toxic side effects. Furthermore, cationic liposomes have been widely researched as effective carriers for gene delivery.^4^ Researchers have discovered that positively charged liposomes, such as those developed by Zhao et al. for bacterial infection treatment, can interact electrostatically with negatively charged bacteria, resulting in effective treatment at the infection site.^5^ Similarly, Ran et al. demonstrated that PEGylated cationic liposome has a more binding affinity toward cancerous cells because of the increase in the negative charge on the angiogenic endothelial membrane due to a higher rate of glycolysis.^6^ As many cancer cells therefore have slightly higher negative surface charge than normal cells, they are more prone to uptake positively charged nanoparticles containing anticancer drugs.^7^ Previous studies have also indicated that such nanoparticles can enhance drug uptake by tumor cells, resulting in increased bioavailability and fewer side effects.^8−10^ However, despite several advantages, the clinical use of cationic liposomes is limited due to rapid clearance by the reticuloendothelial system. Nonetheless, the issue of poor blood circulation can be resolved by modifying the liposome lipid composition. For instance, incorporating a PEG molecule into the liposome can alter its surface properties and improve blood circulation.^11,12^

Even though liposomes are the most studied nanoparticles for drug delivery due to excellent biocompatibility, they often lack significant improvement in therapeutic efficacy. One of the main reasons behind this shortcoming is the inadequate retention of liposomes within target cells as well as the incomplete release of drugs from the liposome vesicles in the desired area. Consequently, there is a pressing need for a formulation that can efficiently deliver higher drug concentrations into tumor cells.

To address this challenge, cationic light-activated thermosensitive liposomes (CLTSLs) have emerged as a potential solution. These liposomes possess dual functionality, combining a positive charge for targeted and enhanced uptake along with thermosensitivity for light-triggered drug release. The CLTSLs use a photosensitizer to induce hyperthermia upon exposure to external light, leading to drug release from the heat-sensitive liposomes.^13,14^

Photoactivated liposomes offer versatility in on-demand drug delivery, with parameters like exposure time, wavelength, beam diameter, and laser intensity^15^ tailored for specific therapeutic purposes, including precise tumor targeting, eye-specific drug delivery,^16^ enhanced antimicrobial treatment,^17^ and wound healing.^18^ An added advantage is the reliance on precise irradiation for accurate targeting, a key benefit of light-sensitive liposomes. This is especially crucial to mitigate the risk of positively charged liposomes binding to nontarget cells, potentially causing harm in other areas of the body. Light activation introduces further control factors, elevating vascular permeability, localized drug release, and modulation flexibility, ultimately reducing toxicity and improving therapeutic outcomes. However, inherent limitations, including limited tissue penetration, phototoxicity, drug loading efficiency challenges, and stability issues, highlight the necessity of developing a robust system to address these challenges in this innovative drug delivery approach. Even though the benefits of these systems are appealing, a meticulous optimization of the liposomes is a crucial step during the development of light-activated systems as these systems require almost negligible passive leakage after administration in order to be effective at the site of action.

The properties of liposomes are heavily influenced by the structural characteristics of the lipids present in their membranes. The length, geometry, nature, and charge of the alkyl chains and headgroups of the lipids, as well as the presence of stabilizers, can all affect the properties of the bilayer.^19^ Therefore, it is crucial to carefully consider the selection of lipids prior to liposome preparation. Additionally, altering the shape of the lipid can greatly impact its behavior since many of its properties are inherently linked to its shape.^20^

In the current study, we used DPPC as a thermosensitive lipid that can form leaky liposomes after exposure to a temperature higher than its phase transition temperature (41 °C). Further, we have used indocyanine green (ICG) as a stimuli-responsive molecule that generates heat after exposure to NIR light at 808 nm. Light-to-heat transformation in the presence of NIR light leads to rapid heating and an increase in the local temperature, allowing the release of loaded drugs. As ICG has already been proven safe in the human body and FDA-approved, it is safe to use in the liposome formulation.^21,22^ Additionally, the NIR light displays better tissue penetration, less scattering, and safer to use as compared to ultraviolet (UV) light.^23^ As the previous studies with this approach have used anionic liposomes,^21^ it would be interesting to look into cationic alternatives to also take advantage of the benefits of the positive surface charge. Combining all of the above functions (cationic surface charge, heat-sensitive lipid composition, ICG dye for light-to-heat conversion) in one formulation makes our designed liposomes extremely promising for effective drug delivery.

This research aims to combine cationic liposomal structures with the photothermal activation approach. Especially, we wanted to study the choice of lipids in detail and develop a good drug delivery system as possible. In previous studies, DOTAP is the most explored cationic lipid to prepare positively charged liposomes. However, it has some limitations in forming stable formulations. Hence, we investigated DPTAP as an alternative option for cationic lipids to form a more stable and effective liposomal system for drug delivery. We believe that our liposomes possess all of the properties required for the adequate performance of nanocarriers based on intravascular drug delivery. The PEGylated lipids ensure more prolonged blood circulation; cationic charge ensures minimum accumulation in nontargeted locations, the stimuli-responsive nature of our liposome serves the function of rapid release on activation and, most importantly, stability. Furthermore, developed liposomes are shown to be stable and do not show any passive leakage at physiological temperature. Numerous attempts were made to develop robust and triggered release cationic liposomes, and a comprehensive list of these trials can be found in the Supporting Information. Out of the various trials conducted, we chose five primary formulations to emphasize the most notable discoveries. Although we will not delve into the specifics of the unsuccessful trials, the Supporting Information provides access to the corresponding data.

## Materials and Methods

### Materials

1,2-Dipalmitoyl-*sn*-glycero-3-phosphocholine (DPPC), 1,2-distearoyl-*sn*-glycero-3-phosphocholine (DSPC), 1-stearoyl-2-hydroxy-*sn*-glycero-3-phosphocholine (Lyso-PC), 1,2-distearoyl-*sn*-glycero-3-phosphoethanolamine-N-[methoxy(polyethylene glycol)-2000] (DSPE–PEG 2000), 1,2-dipalmitoyl-3-trimethylammonium-propane (chloride salt) (DPTAP, 16:0 TAP), and N-[1-(2,3-dioleoyloxy)propyl]-N,N,N-trimethylammonium (DOTAP) were purchased from Avanti Polar Lipids, Inc. (Avanti Polar Lipids, Inc., Alabaster, Alabama). All lipids were stored at −20 °C. All other chemicals were bought from Merck (Merck, Rahway, New Jersey). The HEPES buffer (20 mM HEPES and 140 mM sodium chloride in purified water) and calcein solution (60 mM, 280 mOsm), both adjusted to pH 7.4 with sodium hydroxide (NaOH), were prepared before the liposome preparation.

### Methods

#### Preparation of Light-Activated Liposomes

The ICG and calcein-loaded liposomes were fabricated via a thin film hydration method following the protocol described earlier by Lajunen et al.^21^ This was followed by extrusion to obtain unilamellar vesicles with different lipid molar ratios. Briefly, lipids were dissolved in chloroform to achieve a total lipid concentration of 10 μM, and the dried lipid film was prepared by removing the solvent under vacuum evaporation at 63 °C in a water bath. Further, the thin film was hydrated with calcein solution (60 mM, 280 mOsm, pH 7.4) and 0.322 mg/mL of ICG in 500 μL of HEPES buffer for 1 h. The solution was then extruded through a 100 nm polycarbonate filter using a mini extruder (Avanti Polar Lipids, Inc. Alabaster, Alabama), after which the sample was cooled quickly and stored in a refrigerator (2–8 °C). Further, to remove unencapsulated calcein and ICG, samples were purified by gel filtration through a Sephadex G-50 column with a HEPES buffer. The final lipid and ICG concentration of the purified samples was 1.5 mM and 30 μM, respectively. The list of the prepared liposomal samples is given in Table 1.

**Table 1:** Liposome Compositions and the Physicochemical Properties of the Prepared Liposomes (Represented Data Is Shown with the Mean of Triplicate Measurement with SD)

short forms	lipid composition	molar ratio	size (nm)	PDI	zeta potential (mV)
CLTSL_DSPE_	DPPC:DSPC:DOTAP:Lyso-PC:DSPE	75:5:10:10:4	≥1000		
CLTSL_DSPE-PEG_	DPPC:DSPC: DOTAP:Lyso-PC:DSPE–PEG 2000	75:5:10:10:4	103 ± 23	0.036 ± 0.05	18 ± 0.55
CLTSL_Chol_	DPPC:DSPC:DOTAP:Cholesterol:DSPE–PEG 2000	75:15:10:10:4	131 ± 37	0.084 ± 0.03	14 ± 2.20
CLTSL_DPTAP_	DPPC:DSPC:DPTAP:Lyso-PC:DSPE–PEG 2000	75:15:10:10:4	73 ± 19	0.092 ± 0.03	13.5 ± 0.7
ALTSL	DPPC:DSPC:Lyso-PC:DSPE–PEG 2000	75:15:10:4	112 ± 26	0.039 ± 0.07	–22.5 ± 0.58

(CLTSL_DSPE_, cationic light-activated thermosensitive liposomes; CLTSL_DSPE-PEG_, cationic light-activated thermosensitive liposomes with DOTAP; CLTSL_Chol_, cationic light-activated thermosensitive liposomes with cholesterol; CLTSL_DPTAP_, cationic light-activated thermosensitive liposomes with DPTAP; ALTSL, anionic light-activated thermosensitive liposomes).

#### Characterization of Liposomes

The hydrodynamic diameter of the liposomes was measured with a dynamic light scattering (DLS) automated plate sampler (Zetasizer APS, Malvern Instruments, Malvern, United Kingdom). The size distributions are reported by number PSD (nanometers) and polydispersity index (PdI). Zeta potential was determined using a Zetasizer ZS (Malvern Instruments, Malvern, United Kingdom) using a DTS1070 Zetasizer measurement cell. Three parallel sample runs were measured at 25 °C for all samples.

#### Differential Scanning Calorimetry

The phase transition temperature (Tm) was determined using differential scanning calorimetry (TA DSC2500, TA Instruments, New Castle, United States). Briefly, 20 μL of the unpurified liposome sample was pipetted onto an aluminum pan and sealed with an aluminum lid. The sample and reference pan with the same amount of water were heated using a linear temperature gradient from 25 to 80 °C in a nitrogen environment. The phase transitions were detected as negative endothermic peaks in the baseline-corrected thermographs following analysis with TRIOS software (TA Instruments, New Castle).

#### Temperature-Induced Calcein Release

The heat-induced release of calcein was studied based on the self-quenching phenomena shown by the calcein molecule. At a high concentration, calcein shows reduced emission (inside liposomes) due to aggregation. When it is diluted, such as upon release from liposomes due to heat, calcein again shows strong fluorescence, and a concentration calibration curve can be constructed for lower calcein concentrations. This change in fluorescence is recorded to study the stability of liposomes in the presence of heat. The calcein release was measured over a temperature range of 35–50 °C. Initially, 490 μL of HEPES buffer was heated to the desired temperature in an Eppendorf ThermoMixer (Eppendorf AG, Hamburg, Germany). Once the target temperature was achieved, 10 μL of the purified liposome solution was added to the prewarmed buffer, and the mixture was stirred for 10 min at 300 rpm. The sample was then quickly cooled in an ice bath. The cold control, which contained only 490 μL of buffer, was kept in the refrigerator (2–8 °C) until 10 μL of liposomes was added just before the measurement. Finally, the fluorescence of the calcein (excitation 493 nm, emission 518 nm) was measured using a Varioskan LUX (Thermo Fisher Scientific Inc., Waltham). The calcein release was expressed as a percentage using eq 1
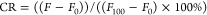
1where *F* is the fluorescence of the sample, *F*_0_ is the background fluorescence measured from the cold control sample, and *F*_100_ is the maximum fluorescence when the content is completely released from the liposomes after the addition of 10 μL of 10% Triton X-100 solution. Triton X-100 completely disrupts the liposomes and causes the full release of the loaded compounds.

#### Light-Induced Calcein Release

Calcein release from liposomes after light activation was studied using an automatic biomedical illumination ML8500 Modulight instrument (ML8500, Modulight Inc., Tampere, Finland). The liposomes were diluted 1:10 (v/v) with HEPES buffer in a clear-bottom, black 96-well plate (Thermo Fisher Scientific Inc., Waltham, MA) and kept at 37 °C for incubation. After a 10 min incubation period, the sample was exposed to an 808 nm laser for 5–120 s with an intensity of 1 W/cm^2^. The control sample on the same plate was protected from light exposure. After laser irradiation, the plate was immediately cooled in an ice bath. Then, the calcein fluorescence (excitation 493 nm, emission 518 nm) was measured with a Varioskan LUX and % calcein release was calculated by using eq 1. The experiment was repeated three times, and the mean percentage of calcein release and the standard deviation were calculated.

#### Molecular Dynamics Simulations of Liposomal Lipid Bilayers

We carried out atomistic molecular dynamics (MD) simulations on two different liposome lipid bilayer membrane systems to investigate the effect of DOTAP and DPTAP on the lipid membrane properties of liposomes. We constructed the simulation systems by using the CHARMM-GUI web-based graphical user interface that can be used to prepare various multicomponent biological systems for molecular dynamics simulations.^24^ All lipid parameters were based on the CHARMM36m force field.^25−27^ We used the same lipid compositions (Table 1) as in the experimental formulations of CLTSL_DSPE-PEG_ and CLTSL_DPTAP_. The total number of lipids in the system was 400. Lipid bilayers were solvated with 50000 water molecules utilizing the TIP3P water model.^28^ To mimic the physiological salt concentration, 150 mM of NaCl was added into the systems, and additional negative counterions were used to obtain charge neutrality for all systems. The steepest descent algorithm with 5000 minimization steps was used to energy minimize the systems before starting simulations. Initially, lipids were simulated up to 1 ns with position restraints on the lipid headgroup and tail carbon atoms with a force constant of 1000 kJ/(mol nm^2^) in the *Z* direction to prevent the separation of lipid monolayer. Consequently, position restraints were removed, and all systems were simulated for up to 1 microsecond. The GROMACS simulation package version of 2020.5 was used to carry out the simulations.^29^ The simulations were carried out using an isothermal–isobaric ensemble with constant NPT. In the isothermal–isobaric ensemble, NPT resembles the number of particles (N), pressure (P), and temperature (T), and all of these parameters are kept constant to achieve conditions closer to laboratory conditions with a flask open to ambient temperature and pressure.

All simulations were coupled to a temperature bath of either 308.15 or 323.15 K, utilizing the Nose–Hoover thermostat with a coupling constant of 1.0 ps.^30^ Lipids and water molecules were coupled to separate the heat baths. Pressure was maintained at 1 bar isotropically using the Parrinello–Rahman barostat with a coupling constant of 5 ps^–1^.^31^ To handle the electrostatic interactions, the particle-mesh Ewald (PME) summation scheme was employed with a real- space cutoff of 1.2 nm.^32^ The Lennard-Jones interaction cutoff was set to 1.2 nm, and the force-switch vdw-modifier was employed starting at 1.0 nm. All bonds with hydrogen were constrained using the LINCS algorithm, and the time step was set to 0.002 ps.^33^

All of the analysis calculations were carried out after 500 ns of simulations, which were needed to reach the area per lipid equilibrium in each simulation system. Order parameters, rotational autocorrelation functions, and angle distributions were determined using the GROMACS analysis tools gmx order, gmx rotacf, and gmx angle, respectively. A block averaging technique was used to calculate the averages and errors for order parameters. After the equilibration time period of 500 ns (confirmed by area per lipid), the simulation trajectory was divided into three 166 ns blocks, for which averages of the chosen quantities were calculated. The average of these averages was calculated and used in the determination of standard deviations. The visual molecular dynamics (VMD) program was utilized to render the figures.^34^

During MD simulations, we chose to model liposomal lipid membranes by using a lipid bilayer model with zero curvature as we believe that the curvature of the liposomes we studied in this research is not high enough to induce considerable deviations in the properties of lipids we are interested in. However, the liposome suspension may contain extremely small liposomes. It is worth mentioning that when the liposome diameter becomes smaller and smaller, especially when reaching diameter values that are comparable to the thickness of the liposome bilayer (4 nm), it might influence calcein release and lipid phase behavior. For instance, the shrinking of liposomes to these very small sizes may tightly pack the inner leaflet, cause asymmetric lipid distribution, and result in high interfacial tensions depending on factors like lipid composition and preparation method.

## Results and Discussion

When formulating stable cationic light-activated thermosensitive liposomes for tunable drug release at the site of action, there are three things that should be focused on: 1. making a robust thermosensitive cationic liposome formulation that would not leak passively during blood circulation;^35,36^ 2. accurate externally controlled triggered release via heat and/or light;^15^ and 3. complete and immediate drug release. Previously reported thermosensitive liposomes in the literature did not simultaneously satisfy all of the conditions mentioned above, making it extremely challenging to develop light-activated thermosensitive liposomes. The situation is complicated since the system requires simultaneously the loading of two molecules (amphiphilic ICG and hydrophilic calcein), a cationic surface charge, and good stability in physiological conditions but rapid content release upon activation. We addressed this issue by fabricating and optimizing these liposomes step by step. A particular focus was on the lipid composition and the effect each lipid choice has on the stability and applicability of the liposomes.

We formulated liposomes with DPPC as a major component; further, DSPC and Lyso-PC were added to adjust the phase transition temperature to a desired level (43 °C) and make them thermosensitive. Then, we used suitable cationic lipids for adjusting the surface charge; finally, ICG was used as a photodynamic molecule to facilitate cargo release upon light activation. In this study, calcein was encapsulated in the liposomes as a model to mimic a hydrophilic drug molecule. The anionic light-sensitive liposomes (ALTSLs) developed by Tatu et al. were used as the basis for developing cationic thermosensitive liposomes, which have been proven stable and suitable for light activation.^21^ In the following sections, we go through the effect of the main lipid components based on our findings one by one.

Several trials were taken to fabricate stable and highly responsive cationic light-activated thermosensitive liposomes (all of the trials are listed in the Supporting Information). Among those trials, we selected the five main formulations listed in Table 1 to highlight the most significant findings. We will not go through the failed trials in detail, but the data are available in the Supporting Information. These experiments are considered as prescreening studies and were used to identify which lipids are the most relevant. In all studies, the liposomes were characterized by measuring the hydrodynamic size, polydispersity index (PDI), zeta potential, and phase transition temperature. Furthermore, liposomes were tested for thermal and light-activated release to study their usability for the triggered content release.

### Influence of Adding DSPE–PEG and DOTAP-Based Cationic Liposomes

The hydrodynamic particle size of all liposomes in the trials mentioned in Table 1 fell within the desired range of 60–120 nm, as determined by dynamic light scattering. However, during hydration of the lipid film in the CLTSL_DSPE_ trial, significant precipitation was observed. The CLTSL_DSPE_ trial contained primary functional lipids required for the purpose of designing cationic light-activated thermosensitive liposomes but lacked DSPE–PEG 2000. A green precipitate was observed after adding a hydrating solution containing ICG and calcein during lipid film hydration. As ICG has both hydrophilic and lipophilic properties, it is likely to be located in the lipid bilayer. This may result in an interaction between free lipids and free ICG molecules, leading to precipitation.^21,37,38^ To evaluate how changing various ratios of lipids in the liposomes can affect the precipitation, we formed liposomes with different molar ratios (trials listed in the Supporting Information). Unfortunately, we observed precipitation in all of the trials. As per the study conducted by Lajunen et al., the polyethylene glycol (PEG) chains in DSPE–PEG 2000 serve as a crucial function in addition to having a stealth effect: PEG provides steric stabilization by wrapping the PEG chains around the free ICG clusters present in aqueous solution and prevents their interaction with other lipids. This results in the formation of uniformed size liposomes with low PDI. Additionally, Zheng et al. showed that the ratio of monomer to dimer ICG was higher in a formulation containing PEG than in ICG solution, depicting that PEG helps avoid aggregation due to ICG in formulations.^39^ Therefore, as per the literature, to tackle the problem of precipitation, we replaced DSPE in CLTSL_DSPE_ with DSPE–PEG 2000. The amount of DSPE–PEG was kept low in order to not shield the cationic charge of the liposomes. Specific attention was paid to the molar ratio of ICG and DSPE–PEG 2000 to ensure there are enough PEG molecules in the formulation to stabilize the ICG. The molar ratio of ICG to DSPE–PEG 2000 was 1:2.5, indicating that there are more PEGs available in the formulation to make the ICG stable. The formulation with DSPE–PEG 2000, CLTSL_DSPE-PEG_, showed a particle size of 103 ± 23 nm with a low PDI value of 0.036, indicating uniform size distribution. Unlike CLTSL_DSPE_, no precipitation was observed during hydration. In this formulation, because of the amphiphilic nature of PEG with hydrophobic ethylene groups in addition to the polar oxygen atoms, ICG places itself in the PEG sheath. Entrapment of ICG among the PEG chains prevents potential interaction of ICG with the lipid bilayer, resulting in the formation of uniformed sized liposomes with low PDI. In addition, CLTSL_DSPE-PEG_ showed a zeta potential of around +18 mV. A positive surface charge can facilitate their entry into cancerous cells.^7^

**Figure 1:**
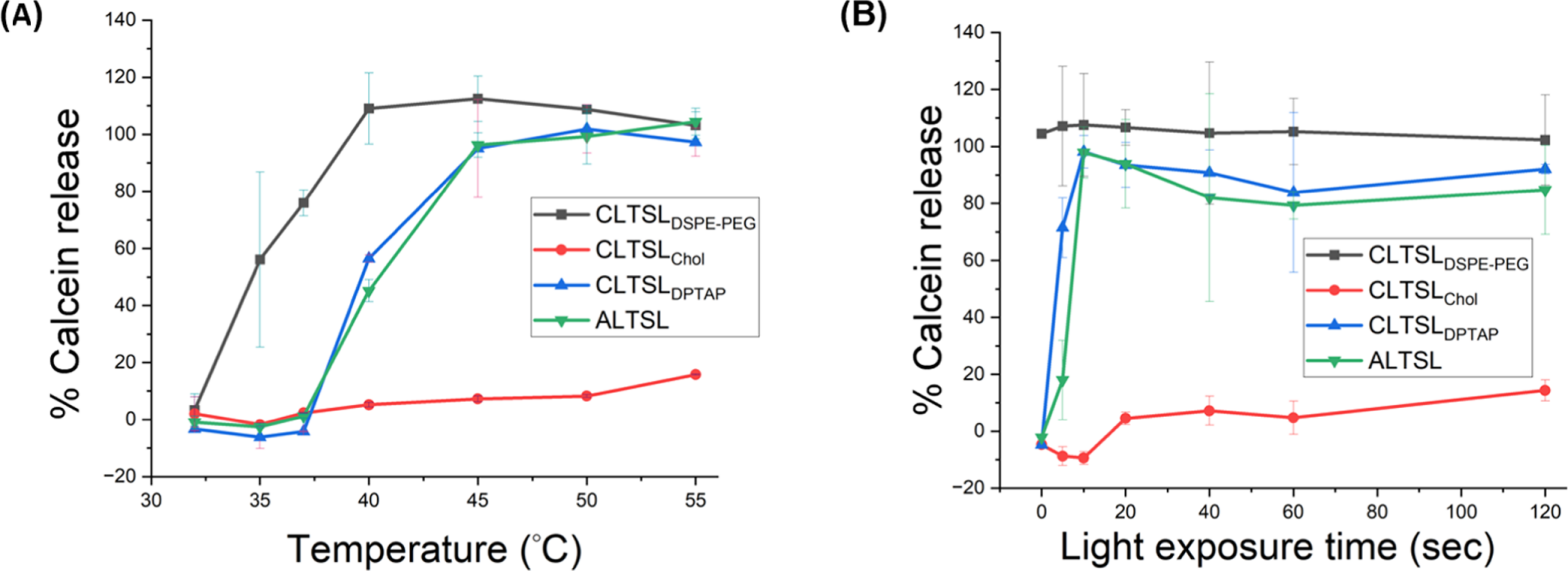
(A) Temperature-dependent release of calcein at different temperatures. The liposome samples were heated for 10 min at varying temperatures to induce the release followed by ice bath treatment. Error bars indicate the standard deviations (*n* = 3). (B) Light-activated calcein release. Samples were preheated at 37 °C and then exposed to an 808 nm 1 W/cm^2^ laser for different durations of exposure time. Error bars indicate the standard deviations (*n* = 3).

**Figure 2:**
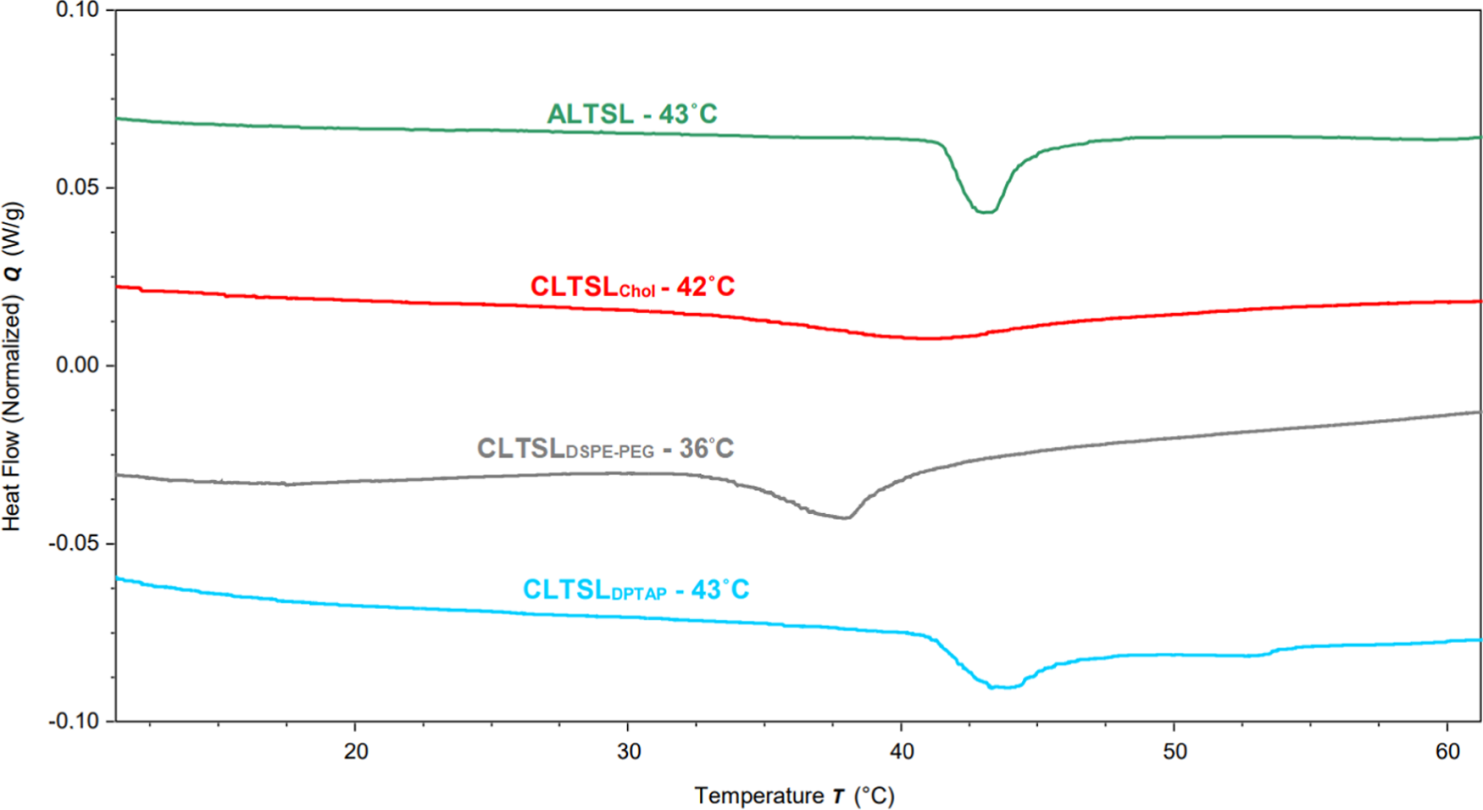
Differential scanning calorimetry (DSC) scans of calcein-loaded liposomes in a HEPES buffer. It is displayed as a heat flow as a function of temperature.

The temperature-induced content release was performed to understand the thermosensitivity of the CLTSL_DSPE-PEG_ liposomes. Temperature and time-dependent calcein release profile from the liposomes at an increased temperature were measured. As shown in Figure 1A, CLTSL_DSPE-PEG_ showed increased calcein release with an increased temperature of up to 55 °C, reaching a 100% release at 40 °C within 10 min of heating. However, CLTSL_DSPE-PEG_ liposomes were also very leaky below these temperatures and released more than 50% of their content at 35 °C (Figure 1A, gray). Importantly, the stability of CLTSL_DSPE-PEG_ liposomes was very poor at physiological temperature, giving an almost 75% release at 37 °C. Additionally, the transition temperature (*T*_m_) of CLTSL_DSPE-PEG_ via DSC revealed that the onset of phase transition was approximately at 32 °C, as shown in Figure 2. This explains the early leakage of liposomes below the physiological temperature. As DOTAP has a low transition temperature, the inclusion of DOTAP in the formulation brings the *T*_m_ of the whole liposome system down.^40^ The *T*_m_ of the liposomal formulation can be adjusted by replacing the DPPC with a high transition temperature lipid such as DSPC. In our trials, this sample was unfortunately very difficult to extrude, and the production of good quality liposomes was not successful.

Furthermore, to study the response of liposomes after light activation, CLTSL_DSPE-PEG_ was exposed to light at 1 W/cm^2^, and content release was calculated after different exposure times, starting from 5 s to 2 min (Figure 1B). Light activation for 5 s was enough to induce complete release from the liposomes. However, the control sample (0 s) (sample protected from light) also released almost all of the content during study as shown in Figure 1B (0 s). These results aligned with thermal release, concluding that the DOTAP-containing liposomes are very unstable at body temperature.

**Figure 3:**
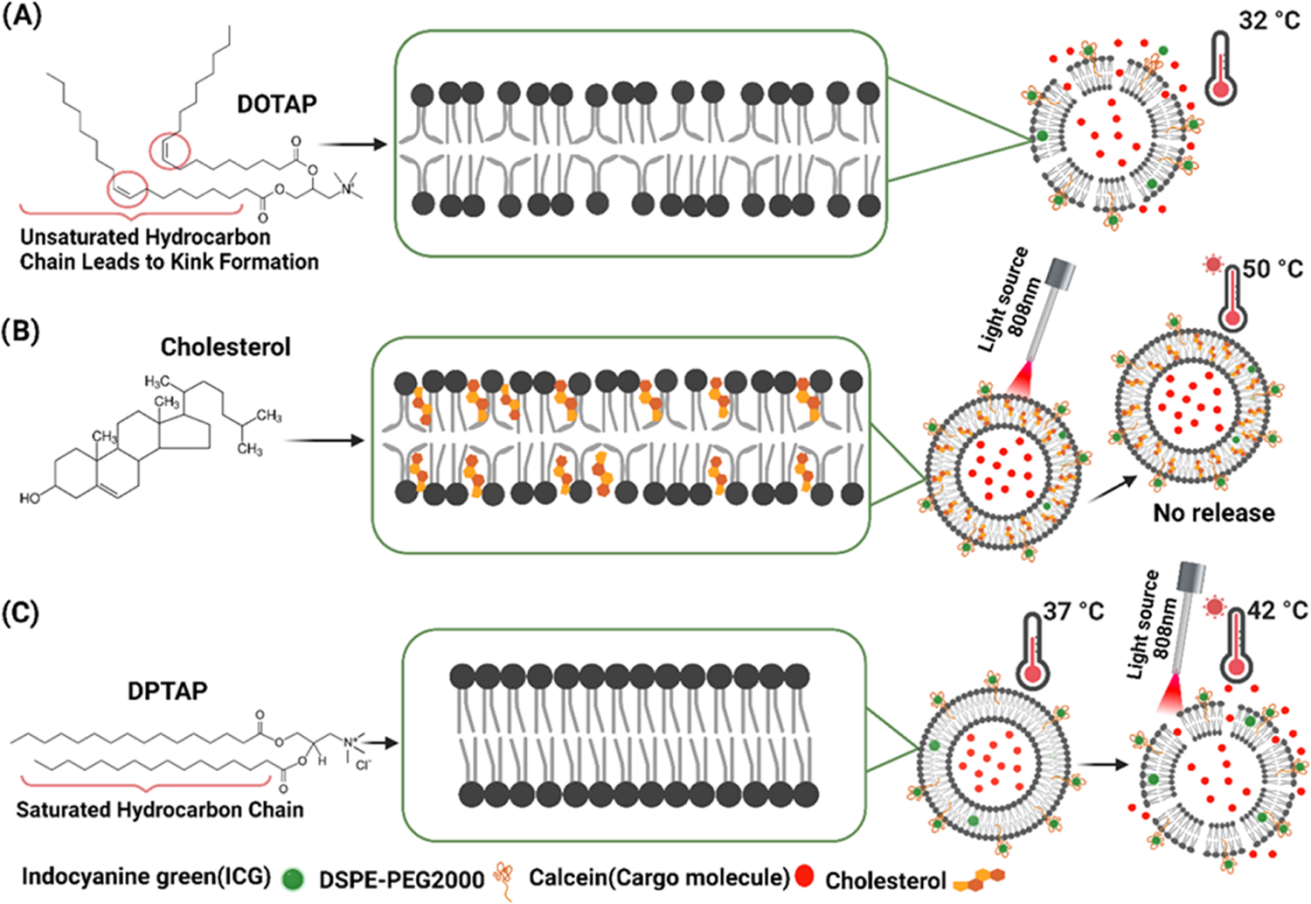
Schematic Illustration of lipid bilayers and their release profile based on light activation. (A) Fluid lipid bilayer with unsaturated lipid (DOTAP) (liposomes were leaky and showed calcein release at 32 °C without light activation). (B) Lipid bilayer with cholesterol (liposomes were very sturdy and did not release calcein even though the temperature reaches 50 °C after light activation). (C) Ordered lipid bilayer with saturated lipid (DPTAP) (liposomes were stable at body temperature (37 °C) and released the calcein after light activation).

Overall, the formulation of cationic light-activated thermosensitive liposomes (CLTSL_DSPE_) lacked the necessary steric stabilization provided by DSPE–PEG 2000, leading to precipitation during lipid film hydration. The addition of DSPE–PEG 2000 in the formulation CLTSL_DSPE-PEG_ resulted in the formation of uniform-sized liposomes. PEG molecules played a vital role in stabilizing ICG by encapsulating it in the PEG sheath, resulting in stable liposomes without aggregation. However, the CLTSL_DSPE-PEG_ liposomes were leaky at subphysiological temperature and had a low transition temperature (Figure 3A). Nevertheless, light activation led to complete release within 5 s, suggesting their potential as a light-activated drug delivery system. The unsuitability of low transition temperature lipids, such as DOTAP, for thermosensitive liposomes was also noted despite their excellent responsive release after light activation.

### Influence of Using Cholesterol

Although CLTSL_DSPE-PEG_ liposomes displayed good size results and showed satisfactory light activation results, they were extremely unstable at body temperature and, hence, unsuitable for drug delivery. It is well-known that using cholesterol affects the phase behavior and the fluidity of lipid membrane. So, to mitigate the leakage problem, we incorporated a rather small fraction (10 mol %) of cholesterol in the formulation. Formulation with cholesterol (CLTSLchol) exhibited a uniform size of around 131 ± 37 nm with low PDI and positive zeta potential around 14 mV. CLTSLchol liposomes showed all desirable colloidal properties as per requirements. Temperature and light-sensitive calcein release profiles for CLTSLchol liposomes are shown in Figure 1A and 1B, respectively. Liposomes were very stable at a temperature below 37 °C, releasing almost nothing at 32 and 35 °C in 10 min. However, CLTSLchol started to release very small amount of calcein at a temperature of around 40 °C, displaying only 15% release at 55 °C. Similarly, these liposomes displayed minimal release of up to 14% after 2 min of light exposure. It was evident from the results that the incorporation of cholesterol into bilayer made liposomes robust. However, CLTSLchol was so sturdy that it cannot be used for light activation (Figure 3B). These findings were further supported by the DSC data presented in Figure 2 (Red). Cholesterol induces a transition from the liquid ordered phase to the gel phase within the lipid bilayer, resulting in a gradual reduction in membrane fluidity beyond the transition temperature.^41^ This transformation is reflected in the DSC analysis, where we observe a broad peak. Notably, previous work by Redondo-Morata and colleagues sheds light on this broader peak observed in cholesterol-containing formulations. At higher cholesterol concentrations, the liposome bilayer’s melting behavior is characterized by two components: a sharp peak, representing the melting of the cholesterol-poor phase, and a broader peak, signifying the melting of the cholesterol-rich bilayer.^42^ The CLTSLchol DSC analysis, as depicted in Figure 2, clearly demonstrates a small, yet broad, peak, indicating an even distribution of cholesterol throughout the lipid bilayer. This even distribution contributes to the exceptional stability of CLTSLchol liposomes. In our pursuit of the optimal cationic liposome formulation, we explored varying cholesterol concentrations. Among these, liposomes containing 5% cholesterol initially demonstrated promising characteristics, showing favorable size, polydispersity index (PDI), and zeta potential results. Unfortunately, these positive attributes were overshadowed by an unexpected instability issue, which manifested after a 24 h storage period at room temperature (data not shown).

Further, we sought alternative approaches to fine-tune our liposome formulations. To explore a new option, we introduced a quaternary amine salt derivative of cholesterol known as DC-cholesterol, aiming to replace the DOTAP in CLTSLchol. This endeavor involved experimenting with two distinct concentrations: 10 and 16% DC-cholesterol. Unfortunately, these liposomes exhibited extreme instability, with significant content leakage occurring within 10 min at 30 °C, making them unsuitable for further testing. These results underscore the intricate and delicate nature of the liposome formulation design.

The literature suggests that adding up to 30 mol % cholesterol significantly decreases the membrane fluidity during the phase transition from the gel phase to liquid phase, making liposome stable.^43−45^ However, recent experiments have revealed that the effect of cholesterol on lipid bilayers is determined by the molecular structure of nearby lipids, particularly the chain unsaturation level, the hydrophobic chain length, and the headgroup composition.^42^ In the current paper, CLTSLchol includes a high concentration of saturated long-chain lipids along with cholesterol, which may be why adding just 10% cholesterol makes liposomes so robust and makes them unresponsive for light-activated release.

In contrast to our findings, some articles suggest that cholesterol is essential for light-activated drug release. Yuan et al. investigated the influence of adding cholesterol in light-activated liposomes. They found that at least 35% cholesterol needs to be added to the bilayer to make the liposomal system responsive to light. Further, they showed that liposomes with 35% cholesterol display increased permeability above a transition temperature. Finally, this study suggests that adding more than 30% of cholesterol to the lipid bilayer increases the fluidity of liposomes, making them release drugs after light activation.^46^ Garcia et al. suggested that liposome containing 40% cholesterol releases the drug more efficiently as compared to a formulation with a lower amount of cholesterol.^46,47^ A possible reason that leads to contradictory findings is variation in the triggering mechanism. It is worth noting that both researchers used gold nanorods in their formulation. The heat generated by gold nanoparticles is higher (75 °C) than that of the ICG molecule (45–50 °C). We believe that the milder conditions with ICG are beneficial for therapeutic applications, but to highlight here, this does lead to challenges in the liposome formulation.

To summarize, the incorporation of cholesterol into the liposome formulation can significantly affect its stability and responsiveness to light activation. Adding cholesterol decreases the fluidity of the membrane, hence resulting in a significant reduction in calcein release.^48^ Further, our findings demonstrate that the addition of just 10 mol % of cholesterol can make the liposomes robust and stable at body temperature. However, this robustness comes at the cost of reduced responsiveness to light activation, which limits their use as vehicles for responsive drug delivery systems. The study emphasized the variation in findings among different researchers, highlighting the importance of considering the nature of neighboring lipids in the composition and heat generation mechanisms when designing liposomal formulations for drug delivery.

### Influence of Using DPTAP over DOTAP

So far, in the studies described above, we have successfully addressed the issue of precipitation and developed a promising thermosensitive formulation using DOTAP(CLTSL_DSPE-PEG_). However, this formulation was very leaky at physiological temperature (37 °C) and too stable with the inclusion of cholesterol, limiting its potential for drug delivery. To resolve this problem, we needed to understand why DOTAP is not suitable in our case and what alternative options can be used to prepare cationic thermosensitive liposomes.

If we look at the structure of the DOTAP lipid, it consists of two double bonds in the hydrocarbon chain (Figure 3A). The presence of the cis double bond results in a kink formation in the hydrocarbon chains of the DOTAP molecule, as shown in Figure 3A.^49^ Because of the kink in the lipid hydrocarbon chain, the lipid tails become disordered within the hydrophobic region of the liposome, which allows for more free lateral movement of lipid molecules within the lipid bilayer, leading to a more fluid and permeable membrane.^50^ Garcia et al. made thermosensitive cationic liposomes using DDAB (unsaturated lipid) as a cationic lipid. As per the results, despite adding 3.35 and 40% cholesterol, the formulations were very leaky and released almost 30% of the drug within 2 h at body temperature.^47^ This indicates that the use of unsaturated cationic lipids leads to the formation of leaky liposomes. Replacing the lipid containing double bonds (DOTAP) with a lipid with no double bonds (saturated lipid) can significantly increase the integrity of the liposomes, making the bilayer less leaky as shown in Figure 3C.

To obtain robust light-activated thermosensitive liposomes, we replaced DOTAP with DPTAP lipid (Figure 3C). DPTAP is cationic in charge and, in contrast to DOTAP, has a saturated hydrocarbon chain and a higher transition temperature of around 49.3 °C. The size of CLTSL_DPTAP_ liposomes was around 73 ± 19 nm. One reason for their smaller size compared to other formulations can be attributed to their tight packing of lipids. As explained above, due to the absence of unsaturated bonds in hydrocarbon chains, DPTAP can be more tightly packed with other lipids compared to DOTAP. Hence, the liposomes formulated with DPTAP are smaller in size than DOTAP liposomes, as shown in Table 1, and had a PDI < 0.1, representing a very high level of particle homogeneity.

The thermal release studies of CLTSL_DPTAP_ were conducted to determine its stability at different temperatures. At a physiological temperature of 37 °C, practically zero percent of calcein was released within 10 min of heating. At 40 °C, CLTSL_DPTAP_ showed around 60% release, while almost 100% calcein was released at 45 °C, as demonstrated in Figure 1A. These results corresponded with the findings from the DSC data displayed in Figure 2, where CLTSL_DPTAP_ showed a transition peak at approximately 43 °C. Importantly, as the onset temperature of the phase transition was observed to occur between 41 and 45 °C, the transition temperatures are feasible to achieve within a few seconds of light activation. The calcein release results after light activation (Figure 1B) are perfectly aligned with the heat release data, showing 71% release in only 5 s of light activation.

Most importantly, CLTSL_DPTAP_ showed almost 100% calcein release at 1 W/cm^2^ after only 10 s of light exposure. This proves the capability of CLTSL_DPTAP_ liposome to release almost all incorporated drugs at the site of action in significantly less time in contrast to comparable formulations available in the literature.^5^ This application can avoid cell damage in healthy cells during light exposure by minimizing the treatment time.

Finally, as the unsaturation in the lipids causes the acyl chains to be more “kinked” and have higher molecular surface area and lower melting point, using unsaturated lipids like DOTAP may cause unwanted leakage in the liposome system. Replacing the unsaturated lipid with saturated DPTAP increases the stability of liposomes while not compromising their light activation properties, unlike liposomes with cholesterol. Using DPTAP has proven to be effective in fabricating stable cationic light-activated thermosensitive and holds immense potential for triggered drug delivery systems. Additionally, CLTSL_DPTAP_ are expected to bind to tumor cells^51^ and deliver drug effectively to the targeted site of action upon light activation. Their dual functionality can be used to facilitate the effective treatment of cancerous tissue and bacterial infections while avoiding toxicity to other cells in the body.

### Molecular Dynamics (MD) Simulations

**Figure 4:**
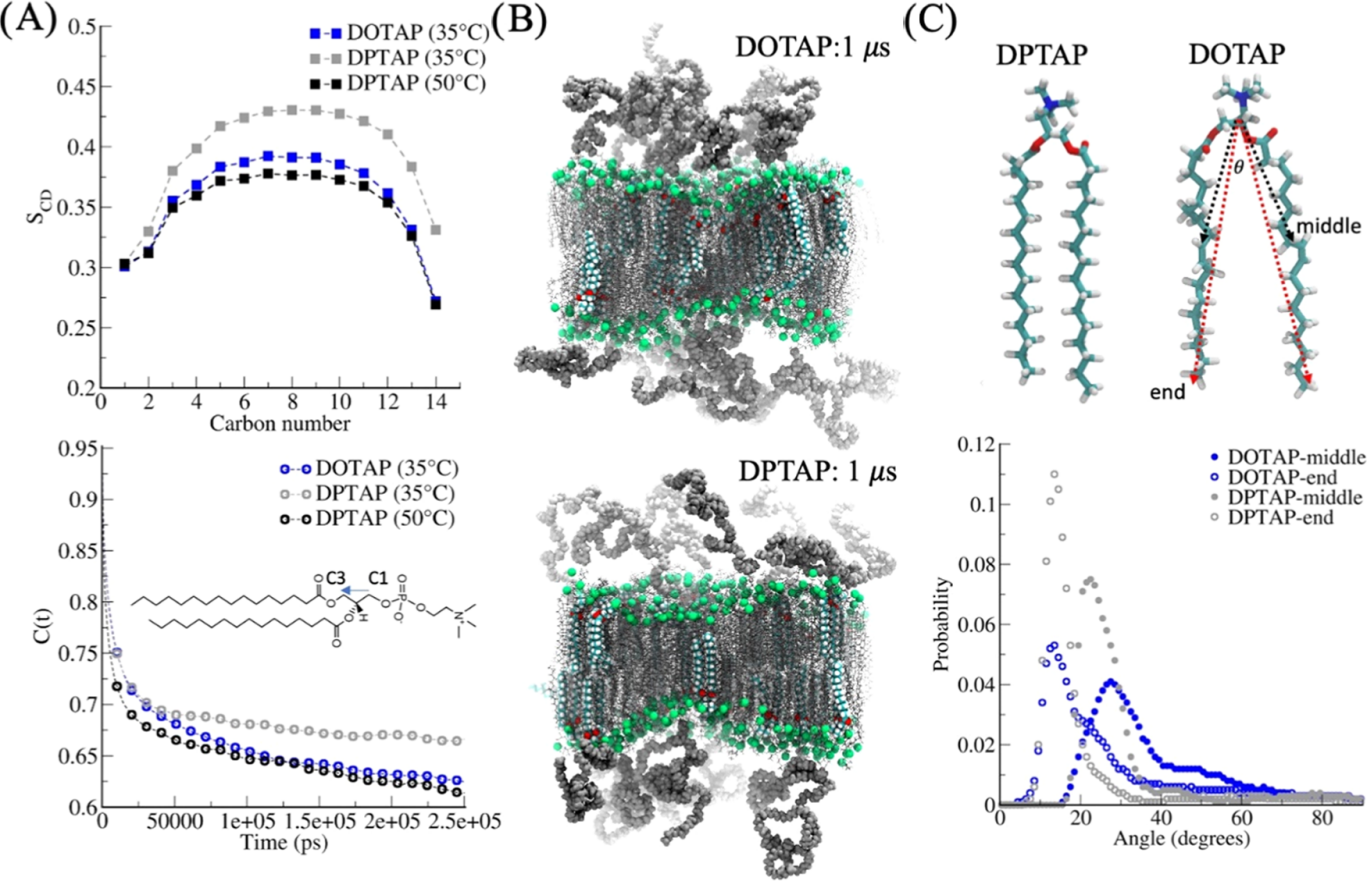
Molecular dynamics simulations of lipid bilayers. (A) Deuterium order parameters for the Sn-1 chain of DPPCs in DOTAP and DPTAP lipid bilayer systems (top). Rotational autocorrelation functions for the C1–C3 vector in the glycerol backbone of DPPC in DOTAP and DPTAP systems (bottom). (B) Snapshots from the end of the DOTAP (top) and DPTAP (bottom) simulations. Phosphorus atoms and PEG chains are colored with green and gray spheres, respectively. DOTAP and DPTAP lipids are rendered with spheres, and different elements are colored as follows: carbon, cyan; oxygen, red; hydrogen, white; nitrogen, blue. Other lipids present in the simulations are rendered with gray sticks (DSPC, DPPC, and DSPE). Water molecules have been removed from the snapshots for the sake of clarity. (C) Forking tendency of DOTAP and DPTAP acyl chains. Vectors used in the analysis (top) and angle distributions (bottom).

To investigate the effect of lipid composition on the dynamical properties of liposomal lipid bilayer, we carried out two atomistic molecular dynamics simulations using the same lipid compositions as in the case of CLTSL_DSPE-PEG_ and CLTSL_DPTAP_ liposomal systems (Figure 4). Both systems were simulated at 35 °C, and additionally, the dynamics of the CLTSL_DPTAP_ system were monitored at 50 °C. We calculated the deuterium order parameters for the Sn-1 chain of DPPC to find out if the replacement of DOTAP by DPTAP (in addition to the relative increase of DSPC) decreases the fluidity of acyl chains. Results in Figure 4A show that the presence of DOTAP lowers the Sn-1-chain rigidity of DPPC at 35 °C when compared to the DPTAP system. As expected, increasing the temperature of the system containing DPTAP to 50 °C decreased the order parameters (Figure 4A). Most likely, DOTAP lipids disturb the packing of neighboring DPPC lipids in a manner that leads to the lowering of the gel-to-liquid crystalline phase transition temperature as shown in DSC results (Figure 2) and previous calorimetric studies with DOTAP–DPPC lipid systems.^52,53^ DOTAP is also known to increase the temperature range over which the gel and liquid crystalline phases coexist.^53^ When the amount of DOTAP was increased in the DPPC bilayer from 0 to 10 mol %, not only the phase transition temperature decreased but also the temperature range where the gel and liquid crystalline phases coexist became wider. Regarding the liposomal formulations in this study, the gel and liquid crystalline phases may also coexist in our DOTAP (∼10 mol %) liposome preparations at 35 °C, leading to strong calcein leakage.

We also investigated how the glycerol backbone dynamics of DPPC is affected by DOTAP as it has been suggested before by Laurdan and DPG fluorescence measurements that the hydrocarbon region of DPPC might be less sensitive to DOTAP-induced disturbance when compared to the glycerol backbone level.^53^ For this reason, we defined a vector between glycerol backbone atoms C1 and C3 and calculated the rotational autocorrelation functions (ACFs) for that vector in each system, (Figure 4A). ACFs indicate that DOTAP changed the glycerol backbone dynamics of DPPC by increasing its spatial rotational dynamics when compared to DPTAP, which can be attributed to the less rigid packing of neighboring DPPC also at the lipid–water interface. However, the magnitude of the effect was not clearly different when it was compared to the effect seen in the case of order parameters. Increasing the temperature from 35 to 50 °C in the case of the DPTAP system accelerated the rotational dynamics of the DPPC glycerol backbone that reached the same level as in the DOTAP system at 35 °C. By inspecting the trajectory snapshots from the end of simulations in Figure 4B, it can be estimated that the acyl chains of DOTAP lipids possess more disordered conformations. A more careful acyl chain angle analysis revealed that DOTAP lipids are more prone to forking, as indicated by the clear shoulders in acyl chain angle histograms near 25 and 45 °C (Figure 4C). It is likely that the presence of splayed DOTAP lipid units can disturb the ordering of saturated lipids the most.

In short, unsaturated DOTAP lipids render the liposomal lipid bilayers more fluidic, at both the acyl chain and lipid–water interface level, when compared to saturated DPTAP lipids. Several splayed DOTAP configurations existed in CLTSL_DSPE-PEG_ bilayers that may contribute more to the disordering of saturated lipid species than the nonsplayed ones. Altogether, these features likely play an important role in lowering the gel-to-liquid crystalline phase transition temperature and leakage of calcein from CLTSL_DSPE-PEG_ liposomes already at a physiological temperature of 37 °C.

## Conclusions

In conclusion, we successfully created a robust and stable cationic light-activated liposomal system for drug release. Through optimization of the liposomal formulation, we were able to achieve rapid drug release upon exposure to near-infrared (NIR) light with minimal passive leakage.

Our research highlights the significant role of lipid structure and properties in the development of liposomes. Our findings demonstrated the critical role of lipid composition in determining the stability and functionality of liposomes. For example, the use of DSPE–PEG can prevent precipitation caused by ICG during the formulation process. The addition of cholesterol in combination with high transition temperature lipids like DSPC and DPPC further improved stability but considerably reduced their ability to release drugs upon exposure to light or heat. In addition, our research highlights the importance of selecting the right cationic lipid. The use of unsaturated and low transition temperature lipids such as DOTAP was found to negatively impact stability and lead to leakiness, whereas a high transition temperature lipid with a saturated structure, such as DPTAP, showed promise in terms of stability and responsiveness to light activation.

Ultimately, the liposomal system developed in this research has numerous potential applications in the delivery of therapeutic agents, particularly for anticancer and antibacterial drugs. The ability to release drugs only when and where necessary can minimize the risk of side effects and maximize the efficacy of the treatment. Finally, our findings underscore the critical importance of lipid structure and properties in the development of liposomes and the potential of these liposomes in the field of on-demand drug delivery systems.

## Supporting Information Available

The Supporting Information comprises a comprehensive list of trials conducted during the study, along with the corresponding observations made throughout these trials (PDF)
