# Supplementary material for: Development of Robust Cationic Light-Activated Thermosensitive Liposomes: Choosing the Right Lipids
Source: Mol Pharm. 2023 Oct 24;20(11):5728–38. doi: 10.1021/acs.molpharmaceut.3c00602 (PMC10630945; doi:10.1021/acs.molpharmaceut.3c00602)
Supplement: Supplementary file 1 — mp3c00602_si_001.pdf [file mp3c00602_si_001.pdf]

Supporting Information

Development of robust cationic light activated thermosensitive liposomes: Choosing the right lipids

Puja Gangurde[1], Mohammad Mahmoudzadeh[1], Zahra Gounani[1], Artturi Koivuniemi[1], Patrick Laurén[1], Tatu Lajunen[1,2], Timo Laaksonen[1,3]*

1. Drug Research Program, Division of Pharmaceutical Biosciences, Faculty of Pharmacy, University of Helsinki, Viikinkaari 5 E, FI-00790 Helsinki, Finland

2. School of Pharmacy, University of Eastern Finland, P.O. Box 1627, FI-70211 Kuopio, Finland

3. Faculty of Engineering and Natural Sciences, Tampere University, FI-33101 Tampere, Finland

Table S1:

Lipid Composition	Molar Ratios	Observations
Trial without DSPE PEG 2000<br>Note: ICG and Calcein (60mM) solutions were used as hydration medium.		
DPPC<br>DOTAP<br>DSPC<br>Lyso PC<br>DSPE	75<br>10<br>5<br>10<br>4	Prominent precipitation was observed after hydration of the lipid film
DPPC<br>DOTAP<br>DSPC<br>DSPE	75<br>10<br>10<br>9	Liposomes were forming large green precipitates
DPPC<br>DOTAP<br>DSPC<br>Lyso-PC<br>DSPE	75<br>10<br>5<br>10<br>4	Large chunks of lipids were observed; However, the mixture was homogenised after vigorous vortexing but was very difficult to extrude.
DPPC<br>DOTAP<br>DSPE	85<br>5<br>10	This trial was taken to see the effect of increasing DSPE concentration and decreasing DOTAP on liposomes, unfortunately the precipitation observed after hydration.
DPPC<br>DOTAP<br>DSPC<br>Lyso-PC	75<br>10<br>5<br>10	We also tried making some formulations without DSPE lipid, to see the effect on the lipid film, unfortunately we found the precipitation after hydration.
DPPC<br>DOTAP<br>DSPC	60<br>20<br>20	Increasing DOTAP concentration leads to more precipitation.

DPPC<br>DOTAP<br>DSPC	50<br>40<br>10	We observed during the hydration that if we increase the DOTAP concentration leads to more precipitation.
Trials with DSPE PEG 2000		
DPPC<br>DOTAP<br>DSPE PEG-2000	85<br>10<br>5	Good trial with no thermosensitive effect. Trial can't be used for light activated release.
DPPC<br>DOTAP<br>DSPE PEG-2000	71<br>25<br>4	Multiple peaks observed after extrusion depicting that higher concentration of DOTAP can cause precipitation (There are not enough DSPE-PEG molecules to avoid DOTAP and ICG interactions)
DPPC<br>DOTAP<br>DSPC<br>Lyso PC<br>DSPE PEG-2000	65<br>20<br>5<br>10<br>4	Unstable formulation (Multiple peaks in size measurement)
DPPC<br>DOTAP<br>DSPC<br>Lyso PC<br>DSPE PEG-2000	57<br>25<br>5<br>10<br>7	Formulation was unstable, formed agglomerated after storage.
DPPC<br>DOTAP<br>DSPC<br>Lyso PC<br>DSPE PEG-2000	75<br>10<br>5<br>10<br>4	Good Trial with satisfactory size (around 100nm) and zeta potential (+ 14mV) . Unfortunately, the trial was leaky at physiological temperature.
DPPC<br>DOTAP<br>DSPC<br>Lyso PC<br>DSPE PEG-2000	75<br>10<br>15<br>10<br>4	Though the transition peak of the formulation was around 42ºC, the formulation was very leaky at 37ºC showing almost more than 50% release in 10min
DPPC<br>DOTAP<br>DSPC<br>DSPE PEG-2000	56<br>10<br>30<br>4	Good trial, Minimal leakage at 37ºC, but the trial was very difficult to extrude and showed no release after light activation.
DPPC<br>DOTAP<br>DSPC<br>DSPE PEG-2000	64<br>2<br>30<br>4	Good transition peak at 44ºC. But did not show any release after or heat light activation.
DPPC<br>DOTAP<br>DSPC<br>Cholesterol<br>DSPE PEG-2000	75<br>10<br>15<br>10<br>4	Formulation did not show any heat or light activation release
DPPC<br>DOTAP	80<br>10	Excluded high transition DSPC and reduced cholesterol concentration very good trial with

Cholesterol<br>DSPE PEG-2000	5<br>5	slight leakage at 35ºC (unsuitable for long term stability)
Batches With Alternative Cationic Lipid		
DPPC<br>DOTAP<br>DSPC<br>DC-Cholesterol<br>DSPE PEG-2000	75<br>10<br>15<br>10<br>4	Formulation was extremely unstable releasing all the content at 30ºC
DPPC<br>DSPC<br>DC Cholesterol<br>DSPE-PEG 2000	54<br>27<br>16<br>3	As per literature suggestion, highly leaky liposomes, releasing almost 90% calcein at 35ºC
DPPC<br>DSPC<br>DSTAP<br>DSPE-PEG-2000	75<br>15<br>5<br>5	Release 31% in 10 min at physiological temperature
DPPC<br>DSPC<br>DSTAP<br>DSPE-PEG-2000	60<br>25<br>10<br>5	Formulation was very stable at 35ºC. Suitable for topical applications, unfortunately leaky at physiological temperature
DPPC<br>DSPC<br>DSTAP<br>DSPE-PEG-2000	37.5<br>50<br>7.5<br>5	Unstable large lipid limps were formed, not suitable for extrusion. Sonication can be done but not tried with this formulation.
DPPC<br>DSPC<br>DPTAP<br>DSPE-PEG 2000	60<br>25<br>10<br>5	Good trial with very minimal leakage at 37ºC and good light activation release but the trial was unstable in long term.
